# Trust and Use of Recommendations for Health Apps Among European Residents: Cross-Sectional Survey

**DOI:** 10.2196/64468

**Published:** 2026-04-09

**Authors:** Mariam Shokralla, Romy Fleur Willemsen, Marise Jeannine Kasteleyn, Niels Chavannes, Esther Talboom-Kamp

**Affiliations:** 1 National eHealth Living Lab, Leiden University Medical Center, Hippocratespad 21, Leiden, 2333 ZD, The Netherlands, 31 639358359; 2 Department Public Health and Primary Care, Leiden University Medical Center, Leiden, The Netherlands; 3 Zuyderland Medical Center, Sittard-Geleen, The Netherlands; 4 Open University, Faculty Management Sciences, Heerlen, The Netherlands

**Keywords:** health apps, recommendations, trust, app assessment, ISO 82304‑2

## Abstract

**Background:**

There is growing recognition of the role of health apps in addressing health care system challenges, yet app quality and evidence vary widely, and consumers have little decision support at the point of download.

**Objective:**

This study aimed to explore which sources of recommendations European residents use and trust when choosing health apps and whether residents support government review and rating of health apps.

**Methods:**

We conducted a cross-sectional online survey (December 7, 2022, to February 16, 2023) in 26 languages targeting residents of the European Economic Area, the United Kingdom, and Ukraine. The survey contained 11 questions covering demographics, types of apps used, sources of advice used and trusted, and views on government review and rating. We included only fully completed responses (N=1228). Descriptive statistics are presented as counts and percentages. For subgroup analyses, we dichotomized trust responses (“I trust” vs other responses) and tested associations with gender (Fisher exact test), age group, and education level (chi-square test); an adjusted significance threshold was applied (*P*<.004) to account for multiple testing. We followed the CHERRIES (Checklist for Reporting Results of Internet E-Surveys) guidelines for reporting online surveys and STROBE (Strengthening the Reporting of Observational Studies in Epidemiology) recommendations for observational studies.

**Results:**

A total of 1228 respondents from 33 countries completed the survey; 1110 (90.4%) reported using one or more health apps. COVID-19 apps (763/1228, 62.1%) and activity apps (737/1228, 60.0%) were the most frequently used, whereas disease management (86/1228, 7.0%), diagnostic (77/1228, 6.3%), and treatment apps (70/1228, 5.7%) were the least used. Sources used to choose apps included family and friends (434/1228, 35.3%), health professionals (412/1228, 33.6%), and government or health authorities (358/1228, 29.2%). The most trusted sources were health professionals (987/1228, 80.4%), pharmacists (750/1228, 61.1%), and government or health authorities (736/1228, 59.9%). No statistically significant differences in trust by gender were observed (all *P*>.0038). Some differences by age and education were observed for select sources (eg, government or health authorities: *χ*^2^_1_=8.546; *P*=.003; family and friends: *χ*^2^=19.133; *P*<.001). Overall, 1060 (86.3%) of 1228 respondents supported government review and rating of health apps (either directly or by commissioning another organization).

**Conclusions:**

In this large multilingual European survey, most respondents reported experience with health apps and placed greatest trust in health professionals, pharmacists, and government or health authorities, yet professional recommendations were used less often than informal sources. There is clear public support for government-led review and rating schemes to guide consumer choice. Efforts to make trustworthy, easy-to-find information available at the point of download and to support health care professionals in recommending high-quality apps could help bridge the gap between trusted and used sources.

## Introduction

### Overview

Health systems in Europe face multiple challenges including aging populations, growing chronic disease burdens, rising costs, and workforce shortages. Digital health tools such as health and wellness apps (“health apps”) are increasingly proposed as part of the response to these pressures [1]. The number of available health apps is large and growing (estimated >350,000 in app stores in 2020), but app quality and the underlying evidence base vary widely [2-4]. Consumers typically have limited reliable information at the point of download beyond app descriptions and star ratings, which do not reliably indicate clinical validity or safety [5-8].

One path to increasing public confidence in health apps is through recommendations from trusted sources (eg, clinicians or health authorities). Experimental and observational research indicates that who recommends an app affects the intention to download it and that clinicians are often trusted but may lack time, training, or trustworthy sources to recommend apps [9-14]. To date, evidence on which sources European residents actually use and trust is limited. This gap motivated the present cross-European survey, carried out as part of the Horizon Europe Label2Enable project, which aims to promote the newly developed [15] CEN-ISO/TS 82304-2:2021 health app quality framework [16].

### Study Objectives

The primary objectives of this study were as follows:

- Identify which sources European residents use to choose health apps
- Identify which sources European residents trust when choosing health apps
- Assess whether residents think the government should review and rate health apps to support choice

The secondary objective was to explore differences in trust by gender, age group, and educational level.

## Methods

### Design and Participants

We conducted a cross-sectional online survey between December 7, 2022, and February 16, 2023, using the Castor electronic data capture system. Eligible participants were residents (aged ≥18 years) of the European Economic Area, the United Kingdom, or Ukraine who could read and respond to a digital survey. No further inclusion or exclusion criteria were applied. The survey was open for 10 weeks and was promoted via the Label2Enable project website, project partners, and social media channels. Because the study was exploratory and descriptive, we did not perform a sample size calculation; however, recruitment and dissemination were monitored to seek diversity across countries, age, gender, and education.

### Survey Instrument

The 11-item survey (Multimedia Appendix 1) covered sociodemographic characteristics (country of residence, year of birth, gender, education level, self-reported health, and informal caregiver status); types of health apps used (17 options); sources of advice used to choose apps (15 options; multiple selections allowed); trust in the same sources (4-point Likert scale); and views on government review and rating (3 options). One open-ended question solicited final thoughts. The English version was drafted in plain language; professional translators translated the survey into 25 other languages (covering 22 of 24 official European Union languages plus Norwegian, Ukrainian, Arabic, and Turkish). Translations were validated by native speakers with expertise in digital health.

### Ethical Considerations

The Medical Ethics Committee of Leiden–Delft–The Hague reviewed the study and waived the need for full ethics approval (reference 223069; November 14, 2022). Participants provided informed consent by completing an online consent statement before starting the survey; they were informed that participation was voluntary, responses were anonymous, and they could withdraw by closing the browser before submission. No personal identifiers were collected. Aggregate data were stored and analyzed in deidentified form. No compensation was offered to participants.

### Translation and Dissemination

Separate language-specific links were hosted on the Label2Enable website, and social media assets were provided to project partners (eg, European Patients’ Forum and EuroHealthNet) to assist dissemination. Response rates were monitored biweekly, and dissemination tactics were adjusted accordingly.

### Statistical Analysis

Only fully completed surveys were included in the analysis (N=1228). Descriptive statistics were presented as counts and percentages; means and SDs were reported for continuous variables (age). To examine associations between demographic variables (gender: male, female, nonbinary, or other; age: 18‐65 vs >65 years; education: low [high school or less or practical training] vs high [bachelor’s, master’s, or doctoral degree]) and trust in different sources, we dichotomized trust responses into “I trust” versus all other responses. For gender, we used the Fisher exact test when expected cell counts were small; for age and education, we used chi-square tests. Because multiple related tests were performed, we applied a Bonferroni-style adjusted significance threshold (α adj=0.05/13=.0038) [17 ]. All statistical analyses were performed in SPSS (version 27; IBM Corp). For qualitative data (open-ended comments) responses were translated to English using DeepL (DeepL SE) and subjected to thematic analysis following the Braun and Clarke six-step approach [18]. We followed the CHERRIES (Checklist for Reporting Results of Internet E-Surveys) guidelines for online surveys and STROBE (Strengthening the Reporting of Observational Studies in Epidemiology) for reporting cross-sectional observational studies.

## Results

### Respondents’ Characteristics

A total of 1228 fully completed responses from 33 countries were included (Multimedia Appendix 1 lists countries and counts). The median age was 47.0 (mean 47.0, SD 14.8) years; 178 (14.5%) were aged 65 years or older. Most respondents were female (787/1228, 64.1%). Education was skewed toward higher education: 1001 (81.5%) of 1228 respondents reported a bachelor’s, master’s, or doctoral degree. Self-reported health was good or very good for 856 (69.7%) of 1228 respondents. A quarter (318/1228, 25.9%) of the respondents provided informal care at least occasionally (Table 1).

**Table 1.** Respondents’ characteristics (N=1228).

Characteristics	Values
Age (y), mean (SD)	47.0 (14.8)
Age (y), n (%)	
65 or older	178 (14.5)
18‐65	1050 (85.5)
Gender, n (%)	
Male	438 (35.7)
Female	787 (64.1)
Nonbinary	3 (0.2)
Education level, n (%)	
High school or less	121 (9.9)
Practical or vocational education	106 (8.6)
Bachelor’s, master’s, or doctoral or similar	1001 (81.5)
Self-reported health status, n (%)	
Poor	76 (6.2)
Fair	206 (24.1)
Good	592 (48.2)
Very good	264 (21.5)
Informal care, n (%)	
I provide informal care every day	106 (8.6)
I provide informal care at least once a week	78 (6.4)
I provide informal care, but not every week	134 (10.9)
I do not provide informal care	910 (74.1)

### Use of Health Apps

Overall, 1110 (90.4%) of 1228 respondents reported using one or more health apps. The most used app types were COVID‑19 apps (763/1228, 62.1%) and activity apps (737/1228, 60.0%). Disease management apps (86/1228, 7.0%), diagnostic apps (77/1228, 6.3%), and treatment apps (70/1228, 5.7%) were the least used (Table 2).

**Table 2.** Types of health apps used by respondents (multiple responses allowed; N=1228).

Type of health apps	Values, n (%)
COVID-19 app	763 (62.1)
Activity app	737 (60.0)
Health insurance app	446 (36.3)
Hospital or clinic app	417 (34.0)
Nutrition app	297 (24.2)
Personal health record app	271 (22.1)
Sleep app	270 (22.0)
Menstruation app	265 (21.6)
Vital signs app	217 (17.7)
Mindfulness app	186 (15.1)
Disease management app	86 (7.0)
Diagnostic app	77 (6.3)
Treatment app	70 (5.7)
Research app	68 (5.5)
Informal caregiver app	19 (1.5)
Other	68 (5.5)
I do not use a health app	118 (9.6)

### Sources of Advice Used to Choose Health Apps

Respondents reported using recommendations from family and friends (434/1228, 35.3%), health professionals (412/1228, 33.6%), and government or health authorities (358/1228, 29.2%) more often than curated sources such as health app libraries (72/1228, 5.9%) or pharmacists (97/1228, 7.9%; Table 3).

**Table 3.** Sources of advice or tips used to choose a health app (multiple responses allowed; N=1228).

Source of advice	Values, n (%)
My family and friends	434 (35.3)
A health professional	412 (33.6)
A government or health authority	358 (29.2)
The App Store or Google Play store	291 (23.7)
A Google search	282 (23.0)
A health professional organization	247 (20.1)
Personal social media posts	183 (14.9)
A patient organization	163 (13.3)
Traditional media	155 (12.6)
A peer support group	129 (10.5)
A health app manufacturer	109 (8.9)
A pharmacist	97 (7.9)
A health app library	72 (5.9)
None of the above	88 (7.2)
Other	54 (4.4)

### Trust in Sources of Advice

When asked which sources they trusted, respondents indicated the highest trust for health professionals (987/1228, 80.4%), pharmacists (750/1228, 61.1%), and government or health authorities (736/1228, 59.9%). Low trust was reported for personal social media posts (65/1228, 5.3%), app stores (144/1228, 11.7%), Google search (118/1228, 9.6%), and health app manufacturers (113/1228, 9.2%; Table 4).

**Table 4.** Trust in advice or tips for a health app from different sources (N=1228).

Source of advice	I do not trust, n (%)	I am not sure if I can or should trust, n (%)	I trust, n (%)	I do not know or have not thought about it, n (%)
A health professional	16 (1.3)	130 (10.6)	987 (80.4)	95 (7.7)
A pharmacist	50 (4.1)	283 (23.0)	750 (61.1)	145 (11.8)
A government or health authority	76 (6.2)	307 (25.0)	736 (59.9)	109 (8.9)
A health professional organization	70 (5.7)	342 (27.9)	688 (56.0)	128 (10.4)
A patient organization (website, brochure, commercial, etc)	71 (5.8)	328 (26.7)	673 (54.8)	156 (12.7)
Family and friends	103 (8.4)	506 (41.2)	512 (41.7)	107 (8.7)
A peer support group	201 (16.4)	540 (44.0)	337 (27.4)	150 (12.2)
A health app library	122 (9.9)	499 (40.6)	335 (27.3)	272 (22.1)
Traditional media	303 (24.7)	635 (51.7)	161 (13.1)	129 (10.5)
The App Store or Google Play store	370 (30.1)	591 (48.1)	144 (11.7)	123 (10.0)
A Google search	358 (29.2)	667 (54.3)	118 (9.6)	85 (6.9)
A health app manufacturer	351 (28.6)	651 (53.0)	113 (9.2)	113 (9.2)

### Differences in Trust by Gender, Age, and Education

The Fisher exact test showed no statistically significant differences in trust by gender across the 13 tested sources at the α adj=.0038 level (all *P*>.0038). Chi-square tests for age and education showed some statistically significant differences for selected sources (eg, government or health authorities: *χ*^2^_1_=8.546; *P*=.003; family and friends: *χ*^2^_1_=19.133; *P*<.001; health app libraries: *χ*^2^_1_=8.249; *P*=.004). However, when applying the adjusted threshold, only *P*≤.0038 was considered statistically significant. Refer to Table 5 for full stratified results and *P* values.

Chi-square analyses indicated that levels of trust in most sources of advice did not differ substantially across age groups (Table 4). Notable exceptions included government and health authorities (*χ*²_₁_=8.55; *P*=.003), family and friends (*χ*²_₁_=19.13; *P*<.001), health app libraries (*χ*²_₁_=8.25; *P*=.004), app stores (*χ*²_₁_=4.12; *P*=.04), Google searches (*χ*²_₁_=5.38; *P*=.02), and health app manufacturers (*χ*²_₁_=4.82; *P*=.03), where significant differences were observed. Similarly, no consistent differences were found between education levels, although significant variation was detected for government and health authorities (*χ*²_₁_=13.02; *P*<.001) and health professional organizations (*χ*²_₁_=8.07; *P*=.005). Full test statistics for all sources of advice are presented in Table 5.

**Table 5.** Trust in sources of advice or tips for health apps, stratified by gender, age, and education.

Trusted advice source	I trust (n=1228), n (%)	Gender			Age group (y)			Education		
		Male, n (%)	Female, n (%)	*P* value	18‐65, n (%)	>65, n (%)	*P* value	Low, n (%)	High, n (%)	*P* value
A health professional	987 (80.4)	350 (79.9)	635 (80.7)	.604	853 (81.2)	134 (75.3)	.11	174 (76.7)	813 (81.2)	.118
A pharmacist	750 (61.1)	262 (59.8)	487 (61.9)	.467	648 (61.7)	102 (57.3)	.421	129 (56.8)	621 (62.0)	.146
A government or health authority	736 (59.9)	272 (62.1)	463 (58.8)	.298	646 (61.5)	90 (50.6)	.003	112 (49.3)	624 (62.3)	<.001
A health professional organization	688 (56.0)	232 (53.0)	454 (57.7)	.247	602 (57.3)	86 (48.3)	.022	108 (47.6)	580 (57.9)	.005
A patient organization	673 (54.8)	225 (51.4)	447 (56.8)	.109	571 (54.4)	102 (57.3)	.392	120 (52.9)	553 (55.2)	.515
Family and friends	512 (41.7)	160 (36.5)	351 (44.6)	.011	463 (44.1)	49 (27.5)	<.001	81 (35.7)	431 (43.1)	.42
A peer support group	337 (27.4)	105 (24.0)	231 (29.4)	.081	289 (27.5)	48 (27.0)	.742	71 (31.3)	266 (26.6)	.152
A health app library	335 (27.3)	120 (27.4)	213 (27.1)	.313	301(28.7)	34 (19.1)	.004	51 (22.5)	284 (28.4)	.071
Traditional media	161 (13.1)	59 (13.5)	101 (12.8)	.424	136 (13.0)	25 (14.0)	.975	28 (12.3)	133 (13.3)	.701
The app store or Google Play stores	144 (11.7)	47 (10.7)	96 (12.2)	.259	130 (12.4)	14 (7.9)	.042	21 (9.3)	123 (12.3)	.199
A Google search	118 (9.6)	33 (7.5)	84 (10.7)	.06	108 (10.3)	10 (5.6)	.02	16 (7.0)	102 (10.2)	.147
A health app manufacturer	113 (9.2)	40 (9.1)	73 (9.3)	.99	104 (9.9)	9 (5.1)	.028	15 (6.6)	98 (9.8)	.134
Personal social media posts	65 (5.3)	21 (4.8)	43 (5.5)	.145	54 (5.1)	11 (6.2)	.798	11 (4.8)	54 (5.4)	.739

### Views on Government Review and Rating of Health Apps

Among the 1228 respondents, a total of 1060 (86.3%) respondents indicated that either the government should review and rate health apps (n=664, 54.1%) or that the government should pay another organization to do so (n=396, 32.2%); 168 (13.7%) opposed government involvement (Table 6).

**Table 6.** Views on whether the government should review and rate health app quality (N=1228).

Response options	Values, n (%)
Yes, I think the government should review and rate health app quality	664 (54.1)
No, but I think the government should pay another organization to review and rate health app quality	396 (32.2)
No, I think the government should not review and rate health app quality and should not pay another organization to review and rate health app quality	168 (13.7)

### Thematic Analysis of Open Comments

Of 1228 respondents, 213 (17.3%) provided free-text comments. Thematic analysis identified 3 main themes [19].

The first theme was exploring apps as a useful part of health care (prescription, reimbursement, and accessibility). Respondents expressed that apps should be prescribed and reimbursed to ensure their continuous improvement and that they should be made accessible to all patients. Health professionals should consider using data and apps to improve their work.

The second theme was identifying the need for quality assessment and transparency (certification and evidence), represented by this quote: “If you want something to work, you have to evaluate it.” Some referred to their role: “I would accept every recommendation first and then research the app myself and test it.” Few saw no need for an assessment: “Bad apps eventually phase themselves out as they increasingly turn out to be bogus.” or “The quality of the health apps can easily be assessed by health care professionals. I think it’s a waste of money to put much effort into this*.*”

The third theme revolved around who should review and rate apps (preference for independent and knowledgeable assessors). Respondents expressed mixed views; some mentioned assessors should be independent with a high ethical standard, have no interest in the app, and have extensive knowledge of both the clinical subject of the app, information and communication technology, and data privacy. Suggestions included a research institute without commercial interests with a high degree of transparency. Others indicated difficulty in identifying any trusted entity to review and rate apps. One comment summarized the 3 themes in one sentence: “Trust in health apps comes from certifications, scientific evidence, and the authorities’ approval and implementation*.”*

## Discussion

### Principal Findings

In this large multilingual European sample (N=1228), the majority reported prior or current use of health apps; COVID-19 apps and activity or wellness apps were the most common. Health professionals, pharmacists, and government or health authorities were the most trusted sources of advice, yet professional recommendations were used less often than informal sources such as family and friends. A strong majority favored government involvement in reviewing and rating apps to support consumer choice.

### Used and Trusted Sources of Advice

Our findings echo prior studies from Australia and Europe that show clinicians are highly trusted but seldom provide app recommendations due to the lack of time, training, and accessible appraisal tools [9-14,20]. One could expect that people would use advice from sources they trust most. However, this discrepancy suggests a limited availability of professional recommendations despite high levels of trust in clinicians. In an Australian study, general practitioners highlighted the lack of knowledge of effective health apps and the lack of trustworthy sources to access high-quality apps as the 2 main barriers to prescribing or providing relevant health app advice [21-23]. A possible solution is to require app stores to display a health app label showing the app’s performance using standardized scales or to create curated health app libraries.

The observed high trust in pharmacists is consistent with literature pointing to pharmacists as potential contributors to digital health counseling [24]. Low public awareness and use of app libraries reflect earlier reports that curated portals are poorly known despite conceptual appeal [25]. Awareness of health app libraries remains limited. For example, one study reported that participants were unfamiliar with the concept of app portals or curated app libraries.[26] Although the idea of curated app repositories indexing government-evaluated and approved apps is appealing, existing initiatives have not yet fully met user needs. The National Health Service for instance, discontinued its central app library and instead integrated recommendations across its website to highlight apps that are already widely used and trusted. Other national initiatives include France’s *Mon Espace Santé* and Germany’s DiGA (*Digitale Gesundheitsanwendungen*; Digital Health Application) directory [27]. Despite the limited uptake among prescribers—only 4% of physicians in Germany had prescribed a DiGA as of early 2022 patient-reported experiences are encouraging: 63% reported a favorable clinical outcome, and 86% indicated willingness to use another DiGA in the future [28,29]. These findings suggest that curated health app libraries, when built on transparent and endorsed evaluation frameworks and made easy to access and navigate, could play an important role in guiding user choice and promoting the safe integration of digital health tools.

### Implications for Policy and Practice

Three implications follow. First, governments and health authorities should consider transparent, evidence-based review and rating schemes (or commissioning trusted independent bodies) and ensure that appraisal results are visible at the point of download. Second, health professionals and pharmacists should be supported with accessible, up-to-date appraisal information and implementation support so they can confidently recommend high-quality apps. Third, further research should test which review and rating mechanisms most effectively change consumer behavior and health outcomes; public preference alone does not establish effectiveness.

### Strengths and Limitations

Strengths include the large multilingual survey, the inclusive plain-language survey instrument, and mixed quantitative and qualitative analysis. A limitation of this study is that the sample was self-selected and skewed toward highly educated respondents, with overrepresentation from some countries (eg, the Netherlands: 403/1228, 32.8%), which limits generalizability. The mean age (47.0, SD 14.8 years) is older than many app user samples and may affect observed patterns of app types and trusted sources. These points are discussed and flagged as limitations. We did not apply poststratification weights to achieve population representativeness; given the exploratory aim, we report unweighted descriptive results but caution against overgeneralization. The thematic analysis was performed on English translations of free-text responses, which may have influenced coding.

### Conclusions

European residents in this large multilingual survey frequently use health apps and place greatest trust in clinicians, pharmacists, and government or health authorities—yet trusted sources are used less often than informal sources. There is clear public support for government-led review and rating or the commissioning of independent organizations to evaluate apps. Policymakers should prioritize transparent, accessible appraisal systems and strategies to support clinicians and pharmacists in recommending high-quality apps.

## Supplementary material

10.2196/64468 Multimedia Appendix 1 The study survey, countries of respondents, and whose advice/ tips do particpants trust to choose a health app.

## Abbreviations

CHERRIES: Checklist for Reporting Results of Internet E-Surveys
DiGA: *Digitale Gesundheitsanwendungen* (Digital Health Application)
STROBE: Strengthening the Reporting of Observational Studies in Epidemiology
